# Long-term Clinical Course after Vitrectomy for Breakthrough Vitreous Hemorrhage Secondary to Neovascular Age-related Macular Degeneration and Polypoidal Choroidal Vasculopathy

**DOI:** 10.1038/s41598-019-57297-8

**Published:** 2020-01-15

**Authors:** Jae Hui Kim, Jong Woo Kim, Chul Gu Kim, Dong Won Lee

**Affiliations:** Department of Ophthalmology, Kim’s Eye Hospital, Konyang University College of Medicine, Seoul, South Korea

**Keywords:** Retinal diseases, Outcomes research

## Abstract

To investigate the long-term clinical course after vitrectomy for breakthrough vitreous hemorrhage secondary to neovascular age-related macular degeneration (AMD) and polypoidal choroidal vasculopathy (PCV). This retrospective study included 45 eyes that underwent vitrectomy due to breakthrough vitreous hemorrhage secondary to neovascular AMD. The patients were divided into 2 groups: neovascular AMD group and PCV group. Within each group, the status of the eye within 6 months after the surgery and that at the final follow-up was identified. The visual acuity at the final visit was additionally compared between the 2 groups. The patients were followed up for a mean period of 39.9 ± 19.4 months after the surgery. In the neovascular AMD group (n = 17), re-bleeding requiring vitrectomy was noted in 4 eyes and extensive scar formation was noted in 6 eyes within 6 months after the surgery. At the final visit, treatment was discontinued due to poor visual outcome in 10 eyes. In the PCV group (n = 28), re-bleeding requiring vitrectomy was noted in 1 eye, and extensive scar formation was noted in 4 eyes within 6 months after the surgery. At the final visit, treatment was discontinued in 8 eyes. The visual acuity at the final visit was significantly better in the PCV group (P = 0.003). The long-term clinical course after vitrectomy for breakthrough vitreous hemorrhage was markedly different between neovascular AMD and PCV, showing significantly better long-term visual outcomes in PCV.

## Introduction

Submacular hemorrhage is frequently encountered in patients with neovascular age-related macular degeneration (AMD) and polypoidal choroidal vasculopathy (PCV)^1^. Although patients with this condition were excluded from key clinical trials^2,3^, subsequent studies have shown that anti-vascular endothelial growth factor (VEGF) therapy is effective even in this condition^4–6^.

Development of dense breakthrough vitreous hemorrhage is one of the complications of large submacular hemorrhage^5,7^. Although vitreous hemorrhage itself does not have a negative impact on fundamental visual function, abrupt and profound deterioration in visual acuity can occur because the hemorrhage blocks the transmission of light to the retina. The hemorrhage also impedes visualization of the retina. Thus, immediate intervention is often necessary to clear the media both to restore vision and to accurately assess the retinal status.

The mainstay of treatment for dense vitreous hemorrhage is vitrectomy. However, vitrectomy is an invasive approach that may be accompanied by some risk of serious complications such as retinal detachment or endophthalmitis^8,9^. Furthermore, if retinal function has been severely damaged due to submacular hemorrhage, the visual acuity may not recover after vitrectomy. Thus, it is necessary to fully discuss the advantages and disadvantages of vitrectomy before the surgery. In this discussion, it may be helpful to provide the patient with information on the postoperative visual recovery, possibility of additional surgery due to recurrent bleeding, and the long-term prognosis.

Previous studies have reported the outcomes of vitrectomy in breakthrough vitreous hemorrhage^10–14^. However, these studies were mainly focused on visual outcomes, especially the short-term outcomes after the surgery. Neovascular AMD and PCV usually require long-term treatment. and it is difficult to expect complete cure. Thus, data on the long-term clinical course are of great value in predicting patient prognosis and determining treatment strategy. To date, the detailed long-term clinical course in this condition has not yet been fully elucidated.

The purpose of the present study was to investigate the long-term clinical course after vitrectomy for breakthrough vitreous hemorrhage secondary to neovascular AMD and PCV, and to identify the differences between the two disorders.

## Materials and Methods

This retrospective, observational, case series was performed at a single center. The study was approved by the Institutional Review Board of Kim’s Eye Hospital and was conducted in accordance with the tenets of the Declaration of Helsinki. Due to the retrospective nature of this study, the need for an informed consent was waived off (Kim’s Eye Hospital IRB, Seoul, South Korea).

### Patients

In the present study, patients with breakthrough vitreous hemorrhage secondary to neovascular AMD and PCV who underwent vitrectomy between January 2013 and December 2017 at our institution were included. The exclusion criteria were as follows: (1) less than 12 months of follow-up after vitrectomy, (2) no available data of indocyanine green angiography (ICGA) or inability to accurately interpret the findings, (3) myopia of −6.0 D or greater and an axial length of 26.0 mm or greater, (4) concomitant retinal vascular disorders (e.g., macroaneurysms, proliferative diabetic retinopathy, and retinal vascular occlusion).

### Examinations

The examination methods used in this study were similar to those used in our previous studies^15,16^. A comprehensive ophthalmological examination, including the measurement of best-corrected visual acuity (BCVA) and a 90-D-lens slit-lamp biomicroscopy evaluation, was performed on all the patients. Fundus photographs were obtained using CX-1 (Topcon, Tokyo, Japan). The fluorescein angiography images were acquired using combined confocal scanning laser ophthalmoscopy and spectral-domain optical coherence tomography (OCT) (Spectralis HRA+OCT; Heidelberg Engineering GmbH, Heidelberg, Germany). ICGA examination was performed at the discretion of the doctor. OCT scans were performed using either the Spectralis HRA+OCT, RS 3000 (Nidek Co., Ltd., Tokyo, Japan), or Spectral OCT (Ophthalmic Technologies Inc., Toronto, Canada).

The diagnosis of PCV was based on the presence of polypoidal lesions with or without branching vascular networks in the ICGA images. For cases in which definite diagnosis of PCV was not possible using ICGA images obtained at the initial diagnosis, ICGA images taken within 3 months after the diagnosis were reviewed as well.

### Treatment and follow-up

Vitrectomy for vitreous hemorrhage was performed using a 23-gauge or 25-gauge sutureless vitrectomy system. Combined cataract operation and/or gas tamponade was performed at the discretion of the doctor. The patients were scheduled to visit the hospital 1 day, 1 week, and 1 month after vitrectomy. Subsequently, the follow-up interval was extended to 2–3 months. In cases without reactivation over a long period of time, the follow-up interval was extended up to 5 months. During the follow-up period, anti-VEGF injections were administered on an as-needed basis using either ranibizumab (0.5 mg/0.05 mL; Lucentis, Genentech, San Francisco, CA), aflibercept (2.0 mg/0.05 mL; Eylea, Regeneron, Tarrytown, NY), or bevacizumab (1.25 mg/0.05 mL; Avastin, Genentech, San Francisco, CA).

In cases of profound visual deterioration, the cost and benefits of the additional treatment were fully discussed with the patients. The treatment was discontinued at the attending doctor’s discretion after discussion with the patient. Patients who discontinued treatment were then followed up every 2–6 months.

### Outcome measures

In all the included eyes, the BCVAs obtained when the hemorrhage was noted, at 1 month after the surgery, and at the final visit were compared.

The primary objectives were to identify the long-term clinical course after surgery for breakthrough vitreous hemorrhage and to reveal any differences in the course between the neovascular AMD group and the PCV group. The status of the eye at early postoperative periods (within 6 months after the surgery) was classified into 4 categories: 1) re-bleeding requiring vitrectomy = vitreous hemorrhage due to re-bleeding from the neovascular lesion for which surgery is indicated; 2) extensive scar formation = development of extensive scar involving the fovea for which further treatment may not be beneficial; 3) underwent anti-VEGF monotherapy = anti-VEGF therapy administered due to presence of fluid or increase in retinal hemorrhage; and 4) no reactivation = absence of fluid or retinal hemorrhage requiring anti-VEGF therapy. The ocular status at final follow-up was classified into 3 categories: 1) treatment discontinued = additional treatment discontinued based on the doctor’s judgment of no potential beneficial effect; 2) ongoing anti-VEGF monotherapy = continuing course of anti-VEGF monotherapy; and 3) no reactivation = absence of fluid or retinal hemorrhage between 6 months after vitrectomy and final follow-up.

The second objective was to evaluate the visual outcomes and to compare the outcomes between the neovascular AMD group and the PCV group: BCVA measured before the surgery, at 1 month after the surgery, and at final visit were compared within each group. In addition, the 3 values were compared between the 2 groups.

Additional analyses were performed to evaluate difference in the characteristics between the neovascular AMD group and the PCV group. The following characteristics were compared; age, sex, hypertension, diabetes mellitus, anticoagulant medication, lens status at the surgery, follow-up period after the surgery. The extent of submacular hemorrhage was defined as the extent measured right before the development of breakthrough vitreous hemorrhage. The maximum extent of submacular hemorrhage was set as 20 disc areas because we believe that hemorrhages exceeding this value may not be accurately measurable. In some cases in which vitreous hemorrhage was the first presentation or fundus images right before the development of vitreous hemorrhage were not available, the extent of submacular hemorrhage could not be measured. Thus, the extent data was presented only as descriptive data, and statistical analysis comparing the value between the 2 groups was not performed.

In PCV cases, the incidence of hyperpigmented spots^15^ was additionally estimated. Hyperpigmented spots were defined by the presence of multiple small, dark-gray or black, pigmented lesions after resolution of hemorrhage.

### Statistics

The data are presented as the mean ± standard deviation where applicable. The BCVA values were converted to logarithm of minimum angle of resolution (logMAR) values for analysis. The visual acuities of counting finger, hand motion, and light perception were converted to logMAR values of 2.6, 2.7, and 2.8, respectively^17^. Statistical analyses were performed using a commercially available software package (SPSS version 12.0 for Windows; SPSS Inc., Chicago, IL, USA). The differences between the 3 time points were analyzed using the repeated-measures analysis of variances and individual comparisons were performed using Bonferroni correction. Differences in characteristics between the 2 groups were analyzed using independent-samples t-test, chi-square test, or Fisher’s exact test. Differences in BCVA between the 2 groups were analyzed using independent-samples t-test with Bonferroni’s correction. P-values <0.05 were considered to be statistically significant.

## Results

Among the 60 eyes, 9 were excluded due to a short follow-up period and 6 were excluded due to lack of ICGA results or inability to accurately interpret the ICGA result. As a result, 45 eyes from 45 patients were included in the study. The mean age was 68.7 ± 8.8 years, and the mean follow-up period from the diagnosis of neovascular AMD to the final visit was 53.2 ± 29.5 months. The mean extent of submacular hemorrhage that resulted in breakthrough vitreous hemorrhage was 17.7 ± 3.2 disc areas in 34 eyes. In the remaining 11 eyes, the extent of hemorrhage could not be accurately measured due to media opacity caused by the vitreous hemorrhage.

During the study period, the mean number of anti-VEGF injections was 7.4 ± 4.7. The type of anti-VEGF drugs used in each patient was as follows: ranibizumab only (8 eyes), aflibercept only (7 eyes), bevacizumab only (6 eyes), and mixed use of 2 or 3 different anti-VEGF drugs (24 eyes). The mean number of anti-VEGF injections was 6.1 ± 4.2 before vitrectomy and 1.3 ± 1.8 after vitrectomy.

The mean timing of surgery was 20.3 ± 23.2 months after the diagnosis of neovascular AMD, Combined cataract surgery was also performed with vitrectomy in 10 eyes, and gas tamponade was performed at the end of the vitrectomy in 2 eyes. The patients were followed up for an additional 32.9 ± 19.4 months after the surgery. During this period, 21 eyes received 2.8 ± 1.7 additional anti-VEGF injections and cataract surgery was performed in 8 eyes. The mean logMAR BCVA was 2.57 ± 0.37 (Snellen equivalents = 20/7430) before the surgery, 1.53 ± 0.87 (20/677) at 1 month after the surgery, and 1.68 ± 0.96 (20/957) at the final visit. When compared with the BCVA before the surgery, the values had significantly improved after the surgery (P < 0.001) and at final visit (P < 0.001).

**Figure 1:**
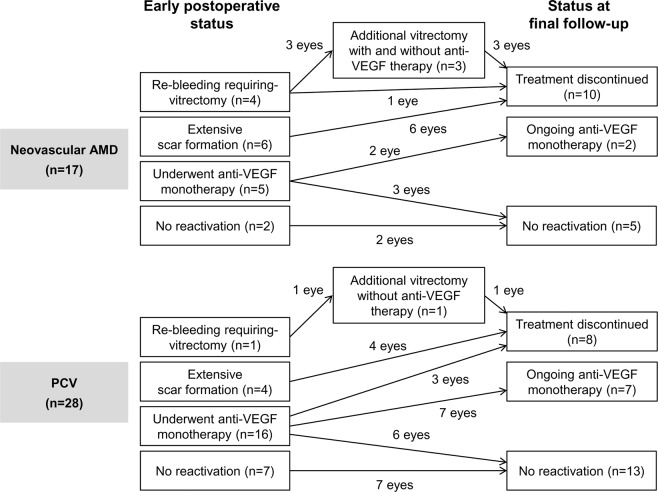
A depiction showing the clinical course after surgery for breakthrough hemorrhage in neovascular age-related macular degeneration (AMD) and polypoidal choroidal vasculopathy (PCV). VEGF = vascular endothelial growth factor.

Seventeen eyes (37.8%) were diagnosed with neovascular AMD, and 28 eyes (62.2%) were diagnosed with PCV. The characteristics of the neovascular AMD and PCV groups are compared in Table 1, and the long-term clinical course in each group is summarized in Fig. 1.

**Table 1:** Comparison of characteristics between the neovascular age-related macular degeneration (AMD) group and polypoidal choroidal vasculopathy (PCV) group.

Characteristic	Neovascular AMD (n = 17)	PCV (n = 28)	P-value
Age, years	74.8 ± 6.6	64.9 ± 7.9	<0.001*
Sex			0.188^†^
Men	10 (58.8%)	22 (78.6%)	
Women	7 (41.2%)	6 (21.4%)	
Hypertension	13 (76.5%)	14 (50.0%)	0.079^‡^
Diabetes mellitus	4 (23.5%)	5 (17.9%)	0.711^†^
Use of anticoagulants	5 (29.4%)	9 (32.1%)	1.000^‡^
Lens status at the surgery			1.000^†^
Phakia	14 (82.4%)	24 (85.7%)	
Pseudophakia	3 (17.6%)	4 (14.3%)	
Extent of submacular hemorrhage**	18.5 ± 1.8	17.1 ± 3.9	
Follow-up period after the surgery	39.0 ± 19.4	29.3 ± 18.8	0.102*

The data are presented as the mean ± standard deviation or No. (%) where applicable.

*Statistical analysis with the independent-samples t-test.

^†^Statistical analysis with the Fisher’s exact test.

^‡^Statistical analysis with the chi-square test.

**Values from 14 eyes of the neovascular AMD group and 20 eyes of the PCV group.

**Figure 2:**
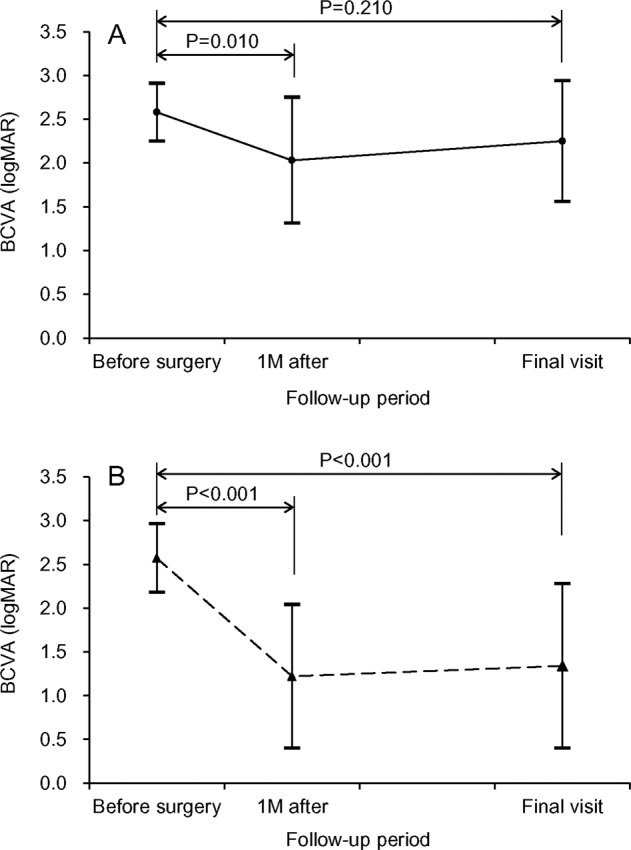
Changes in the logarithm of minimal angle of resolution (logMAR) best-corrected visual acuity (BCVA) in the neovascular age-related macular degeneration group ((**A**) n = 17) and the polypoidal choroidal vasculopathy group ((**B**) n = 28). Statistical analysis was performed using repeated-measures analysis of variances with a Bonferroni’s correction.

In the neovascular AMD group, early re-bleeding requiring vitrectomy developed in 4 eyes (23.5%); 3 eyes underwent additional vitrectomy with or without anti-VEGF therapy and the remaining 1 eye discontinued treatment. Extensive scar formation was noted in 6 eyes (35.3%); treatment was discontinued in all 6 eyes. Anti-VEGF monotherapy was performed after the surgery in 5 eyes (29.4%); reactivation of the lesion was not noted in 3 of these eyes after 1 to 2 additional anti-VEGF injections. Active anti-VEGF therapy continued throughout the follow-up period in 1 eye. In the remaining 2 eyes (11.8%), there was evidence of reactivation after the surgery. The BCVAs measured before the surgery, at 1 month after the surgery, and at final visit were 2.58 ± 0.33 (20/7603), 2.03 ± 0.72 (20/2143), and 2.25 ± 0.69 (20/3556), respectively (Fig. 2A).

**Figure 3:**
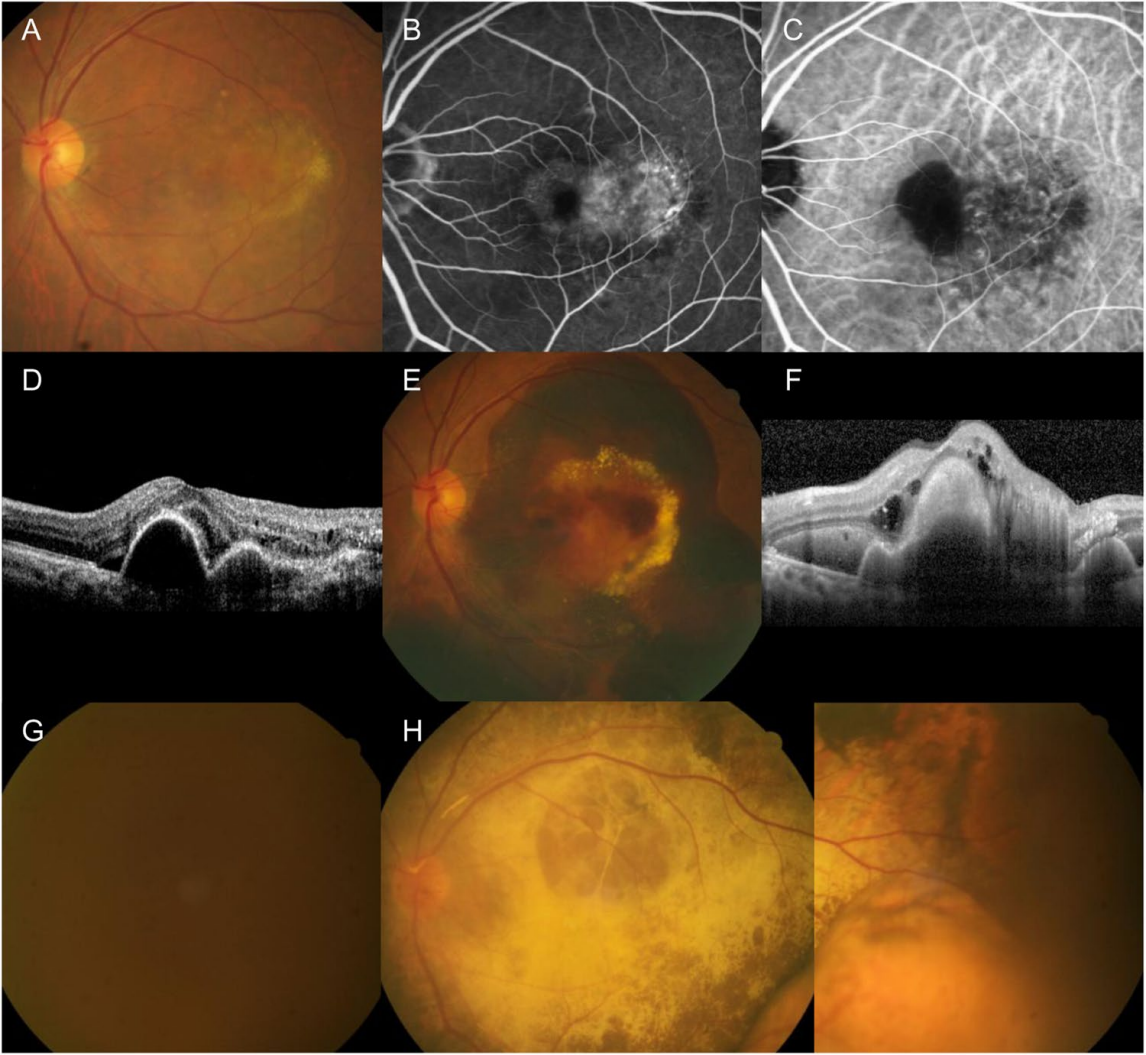
Clinical course of a 73-year-old patient. The patient was diagnosed with neovascular age-related macular degeneration (AMD) (**A**–**D**) and treated with anti-vascular endothelial growth factor (VEGF) therapy. Development of submacular hemorrhage was noted 9 months after diagnosis (**E**,**F**), and the patient received 2 additional anti-VEGF injections. However, development of breakthrough vitreous hemorrhage was noted one month after submacular hemorrhage and the visual acuity declined to hand motion (**G**). One month after vitrectomy (**H**), extensive subretinal scarring was noted and the visual acuity was still measured as hand motion. Further treatment was discontinued after discussions with the patient. A, E, G, H = Fundus photographs; B = fluorescein angiography image; C = indocyanine green angiography image; D, F = optical coherence tomography image.

When compared to the value before the surgery, the BCVA measured at 1 month had significantly improved (P = 0.010), whereas there was no significant difference in the value at the final visit (P = 0.210). Before the surgery, none of the eyes exhibited 20/200 or better BCVA. At 1 month and at the final visit, 3 eyes (17.6%) and 2 eyes (11.8%), respectively, exhibited 20/200 or better BCVA. None of the eyes achieved 20/40 BCVA after the surgery. Figure 3 shows the representative clinical course of an eye with neovascular AMD.

In the PCV group, early re-bleeding requiring vitrectomy developed in 1 eye (3.6%); the eye underwent additional vitrectomy but eventually, treatment was discontinued due to the poor visual outcome. Extensive scar formation was noted in 4 eyes (14.3%); treatment was discontinued in all 4 eyes. Anti-VEGF monotherapy was performed after the surgery in 16 eyes (57.1%); the treatment was eventually discontinued due to poor visual outcome in 3 eyes and reactivation of the lesion was not noted in 6 eyes after 1 to 2 additional anti-VEGF injections. Active anti-VEGF therapy continued throughout the follow-up period in the remaining 7 eyes. In 7 eyes (25.0%), there was evidence of no reactivation after the surgery. The BCVAs measured before the surgery, at 1 month after the surgery, and at the final visit were 2.57 ± 0.39 (20/7430), 1.22 ± 0.82 (20/331), and 1.34 ± 0.94 (20/437), respectively (Fig. 2B). When compared to the value before the surgery, the BCVA had significantly improved at 1 month (P < 0.001) and at the final follow-up (P < 0.001).

**Figure 4:**
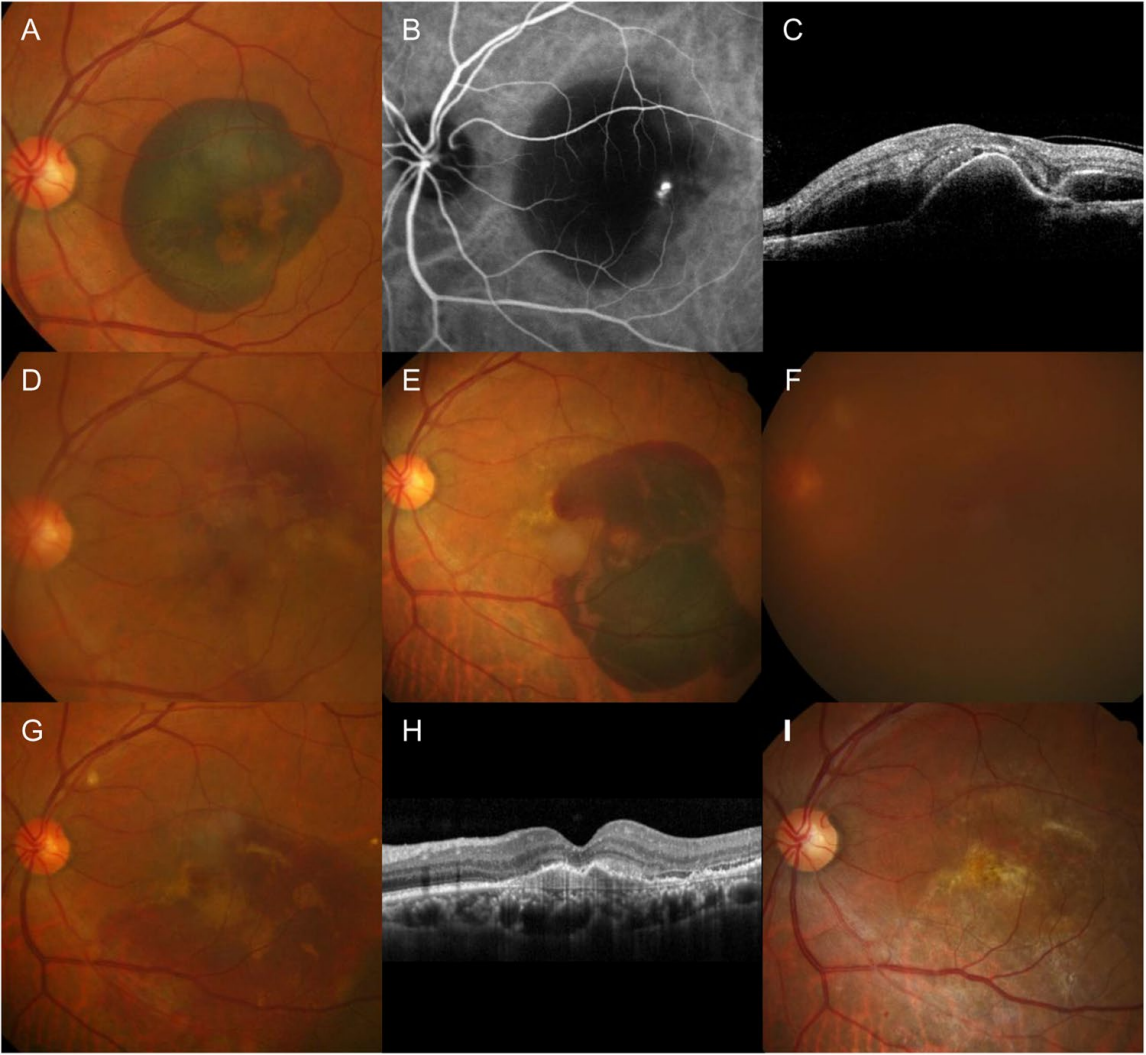
Clinical course of a 66-year-old patient who was diagnosed with polypoidal choroidal vasculopathy. The submacular hemorrhage was noted as an initial presentation (**A**–**C**). After anti-vascular endothelial growth factor (VEGF) injections, the hemorrhage was resolved. Sixteen months later, submacular hemorrhage recurred (**E**) and the patient received 2 anti-VEGF injections. The development of breakthrough vitreous hemorrhage was noted one month after submacular hemorrhage and the visual acuity declined to hand motion (**F**). One month after vitrectomy (**G**,**H**), the visual acuity improved to 20/100. The patient received 6 additional anti-VEGF injections and cataract surgery during the 42-month follow-up period after vitrectomy. At the final visit (**I**), the visual acuity was maintained as 20/100. A, D–F, G,I = Fundus photographs; B = indocyanine green angiography image; C, H = optical coherence tomography image.

Before the surgery, no eyes exhibited 20/200 or better BCVA. At 1 month, 16 eyes (57.1%) exhibited 20/200 or better BCVA and 3 of them exhibited 20/40 or better BCVA. At the final visit, 14 eyes (50.0%) exhibited 20/200 or better BCVA and 4 of them exhibited 20/40 or better BCVA. Hyperpigmented spots were noted in 20 eyes (80%) after resolution of submacular hemorrhage. Figure 4 shows the representative clinical course in an eye with PCV.

BCVA before the surgery was not different between the neovascular AMD group and the PCV group (P = 1.000). However, BCVA at 1 month after the surgery (P = 0.006) and that at the final visit (P = 0.003) were significantly better in the PCV group than in the neovascular AMD group.

In one eye with PCV, retinal detachment was noted at 11 months after the surgery and was successfully treated with scleral encircling, vitrectomy, and gas tamponade. Endophthalmitis was noted in none of the included eyes. In all 20 eyes with discontinued treatment, the visual acuity was worse than 20/400 at the time of determining discontinuation of the treatment.

## Discussion

Vitrectomy for eyes with breakthrough vitreous hemorrhage is known to have a short-term benefit in improving vision. In the study by Chaudhry *et al*., 2 or more lines of visual improvement were noted in 5 of the 6 eyes^10^. Jung *et al*., showed that the mean logMAR BCVA significantly improved from 2.79 before the surgery to 1.61 at 2 months after the surgery^12^. The studies by Lin *et al*. (the mean BCVA improved from 2.63 to 1.62 at 3 months after the surgery)^13^ and by Narayanan *et al*., (the mean BCVA improved from 2.69 to 1.65 at 1 month after the surgery)^14^ also showed similar outcomes. However, considering the degree of postoperative visual acuity itself, the results were somewhat disappointing with better than 20/200 BCVA achieved in only 2 of the 5 eyes (40.0%)^10^, 6 of 24 eyes (25.0%)^12^, and 5 of 17 eyes (29.4%)^13^. In addition, in the study by Roufail and Polkinghome, visual improvement was not noted in 4 of the 5 eyes after the surgery^11^, indicating the profound damage to fundamental visual function in these eyes.

To date, limited information is available regarding the long-term postoperative clinical course in eyes that received vitrectomy for breakthrough vitreous hemorrhage. In the study by Lin *et al*. with PCV^13^, relatively favorable long-term visual outcomes were noted, with the mean logMAR BCVA slightly improving from 1.62 at 3 months after the surgery to 1.43 at a mean of 25.2 months after the surgery. There was no recurrent vitreous hemorrhage during the follow-up period. In previous studies such as the study by Jung *et al*.^12^, which included both neovascular AMD and PCV, the mean logMAR BCVA was 1.61 at 2 months after the surgery and 1.72 at 15.6 months after the surgery. During postoperative follow-up, recurrent vitreous hemorrhage was noted in 4 eyes and retinal detachment developed in 1 eye. The treatment was discontinued in 3 of 12 eyes with neovascular AMD due to poor treatment outcomes. The functional outcomes were better in PCV than in neovascular AMD^12^.

The results of the present study were generally comparable with those of previous studies. Significant improvement in visual acuity was noted after the surgery for breakthrough vitreous hemorrhage in both the neovascular AMD and PCV groups.

In the present study, we focused on the changes in the status of the eye after surgery. The results can be summarized as follows.

1. The prognosis of eyes experiencing re-bleeding requiring vitrectomy was very poor, with the treatment being eventually discontinued in all these eyes.
2. If there is no extensive scar formation or early re-bleeding requiring vitrectomy, the eye may maintain a stable state for a long period of time.
3. In some cases, there was no reactivation immediately after the operation. In other cases, treatment was required immediately after the operation but reactivation was not noted thereafter.
4. Both the short-term and the long-term clinical course markedly differ between neovascular AMD and PCV. The visual outcome was significantly better in PCV than in neovascular AMD. In addition, the incidence of treatment discontinuation was much lower in PCV than in neovascular AMD.

Successful vitrectomy can restore most of the vision loss due to breakthrough hemorrhage. The retinal status and nature of underlying neovascular lesion are main influencing factors of the clinical course after vitrectomy. Moreover, subretinal hemorrhage is associated with poor visual prognosis in neovascular AMD^18^. Studies using animal models reported diffuse degeneration of the retinal layers after subretinal hemorrhage^19–21^. A recent *in vivo* study reported marked thinning of the outer retinal layers after resolution of the hemorrhage^22^. Subretinal hemorrhage is a known risk factor of fibrotic scarring in PCV^23^. In the present study, we observed extensive scar formation in 22.2% of the patients after vitrectomy, which may have originated from the negative impact of subretinal hemorrhage on the retina. The eyes with extensive scar showed poor prognosis, which is an expected finding since the scar is a morphologic factor associated with poor visual outcome^24^. The finding of poor prognosis in the eyes with re-bleeding requiring vitrectomy may be interpreted similarly. Repeat occurrence of breakthrough hemorrhage suggests recurrence of the subretinal hemorrhage, which may lead to cumulative retinal damage.

One interesting finding of the present study is that reactivation was not noted after 6 months in 18 of 45 eyes (40.0%), indicating stabilization of the lesion in these eyes. In addition, the proportion of eyes without showing reactivation was relatively higher in the PCV group (46.4%) than in the neovascular AMD group (29.4%). Previous studies have shown that PCVs presenting with submacular hemorrhage may show a different clinical course from those without hemorrhage^15,16,25^. One of the distinguishing characteristics is that the incidence of reactivation of the lesion is markedly low in these cases^16,25^. In particular, this trend is more pronounced in eyes showing hyperpigmented spots, which suggests degeneration of the retinal pigment epithelial layer and outer retinal layers^15^. We previously suggested that decreased functioning of the damaged retinal tissue is one of the causes for the low incidence of reactivation after submacular hemorrhage^16^. In the present study, the included eyes usually exhibited large-sized submacular hemorrhage. In addition, hyperpigmented spots were noted in the majority of PCV cases. We postulate that the large amount of hemorrhage induced extensive damage to the retinal tissue, resulting in decreased nutrient and oxygen demand. Eventually, secretion of VEGF from the tissue may have decreased and subsequently contributed to low incidence of reactivation.

The markedly different clinical course after vitrectomy between neovascular AMD and PCV may be mainly due to the differences in the two underlying neovascular lesions. Whether PCV is a subtype of neovascular AMD remains controversial^26,27^. However, anti-VEGF treatment originally developed to treat neovascular AMD is also effective in PCV^28^, which suggests that the two disorders have a common main pathophysiology. In general, PCV shows a relatively benign natural course, and associated scarring develops in a limited proportion of patients^29,30^. In our study, the PCV group showed a markedly lower incidence of extensive scar formation than did the neovascular AMD group. Moreover, previous studies indicated that the overall treatment outcomes in hemorrhage cases were better in PCV than in neovascular AMD^5^. The reason for this difference in treatment outcomes is unclear but could be explained based on the phenomena that PCV generally develops in relatively younger patients as compared to neovascular AMD^1^, and age-related pathologic changes, such as drusen and pseudodrusen occur less frequently in PCV^31^. We postulate that the retina was relatively healthier in PCV versus neovascular AMD and more resistant to damage caused by the hemorrhage.

Our results suggest that the clinical course within 6 months after the surgery may provide important information for predicting long-term prognosis. More specifically, if extensive re-bleeding or scar formation is not noted in the early postoperative period, the patient should be counseled that he or she can preserve vision through long-term active treatment. In addition, when discussing the treatment for breakthrough vitreous hemorrhage with the patient, PCV patients should be informed that the likelihood of a good long-term prognosis is high, and the patients should be encouraged to receive active treatment. Conversely, patients with neovascular AMD should be advised that the treatment outcomes can be more unfavorable than expected.

The strength of the present study is that it is the first to evaluate the detailed long-term clinical course after vitrectomy for breakthrough vitreous hemorrhage. In addition, this study was conducted on the largest sample size with the longest follow-up among studies on similar topics. With an increase in the population of patients undergoing long-term active treatment for neovascular AMD^32^, it is possible that the number of patients undergoing vitrectomy due to breakthrough hemorrhage may also increase. It is our understanding that this study may provide useful information when discussing disease prognosis and potential therapeutic options with the patients.

However, the present study also has several limitations. First, anti-VEGF treatment after vitrectomy was performed on an as-needed basis. Thus, the results of the present study may not be valid for patients who were treated with proactive regimens such as the treat-and-extend regimen after the surgery. In addition, strict monthly follow-up was not performed, and the treatment was discontinued in 44.4% of the included eyes. Thus, some of our patients may have been undertreated. Due to retrospective study design, we were unable to identify the reason for treatment discontinuation in each patient. In addition, no strict guideline is available for the discontinuation of treatment. However, considering that the patients showed poor visual acuity at the time of discontinuing the treatment, the decision to discontinue treatment was mainly determined by the doctor’s judgement that no beneficial effects could be achieved by additional treatment and not by the patient’s request in those with meaningful vision. Nevertheless, our study has a limitation of treatment discontinuation without known reason. Further studies with uniform guideline for treatment discontinuation are needed to assess long-term treatment outcomes in similar conditions. Lastly, only vitrectomy to clear the media was performed in most of the eyes. Thus, the results of the present study may not accurately reflect the outcomes of patients who underwent additional treatment such as gas tamponade or tissue plasminogen activator injection.

In summary, vitrectomy was an effective method to restore vision in patients with breakthrough vitreous hemorrhage secondary to neovascular AMD and PCV. However, the clinical course after vitrectomy was markedly different between neovascular AMD and PCV, resulting in significantly different long-term visual outcomes between the 2 groups. The clinical course during early postoperative period may be prognostic of the long-term treatment outcome.

## Data Availability

The datasets generated during and/or analysed during the current study are available from the corresponding author upon reasonable request.
